# Nausea and Vomiting in Pregnancy: Prevalence, Clinical Characteristics, and Management Findings from a Prospective Italian Multicenter Cohort Study

**DOI:** 10.3390/life16030404

**Published:** 2026-03-03

**Authors:** Nicola Colacurci, Giuseppe Bifulco, Mario Fordellone, Gaetano Munno, Dario Colacurci, Marco La Verde

**Affiliations:** 1 Obstetrics and Gynecology Unit, Department of Woman, Child and General and Specialized Surgery, University of Campania “Luigi Vanvitelli”, 80138 Naples, Italy; nicola.colacurci@yahoo.it (N.C.); gmm9401@gmail.com (G.M.); 2 Department of Public Health, School of Medicine, University of Naples Federico II, 80131 Naples, Italy; giuseppe.bifulco@unina.it (G.B.); dario.colacurci@unina.it (D.C.); 3 Statistic Unit, University of Campania “Luigi Vanvitelli”, 80138 Naples, Italy; mario.fordellone@unicampania.it

**Keywords:** nausea and vomiting, pregnancy, Pregnancy-Unique Quantification of Emesis, hyperemesis gravidarum, quality of life, maternal medicine

## Abstract

Objective: Nausea and vomiting in pregnancy (NVP) have a negative impact on quality of life and nutritional status and may progress to hyperemesis gravidarum (HG). We explored the incidence, severity, clinical evolution, and management of NVP. Methods: In accordance with the Italian Society of Gynecology and Obstetrics (SIGO), we conducted a multicentric prospective cohort study at eighteen Italian hospitals, from October 2022 to November 2024. We enrolled pregnant women before 13 weeks of gestation. The severity of NVP and its management were assessed during pregnancy. Results: A total 890 pregnant participants completed the follow-up. NVP prevalence was 70.0% and was classified as 54.4% mild, 42.3% moderate, and 3.2% severe according to the PUQE score; 2.4% required hospitalization. Severe NVP was more frequent in multiparous women (90.0%; *p* < 0.001); NVP history was independently associated with NVP recurrence, OR 3.20 (2.12–4.83; *p* < 0.001). NVP cases showed a low rate of smoking (3.9% vs. 7.1%; *p* = 0.04). After the first consultation, pharmacological treatment, primarily doxylamine–pyridoxine, was prescribed to 50.7% of mild, 67.0% of moderate, and 50.0% of severe PUQE scores. Dosages of ≥3 capsules/day were common in moderate (51.0%) and severe (70.0%) NVP cases (*p* < 0.001). By the second visit, continuation of therapy did not differ among PUQE classes, although reasons for discontinuation varied (*p* < 0.001). By the third visit, therapy continuation dropped to 32.1% in moderate cases (*p* = 0.03). Conclusions: NVP is a common disorder in pregnancy, with a predominance of mild and moderate symptoms. Prior NVP increases the recurrence risk threefold. Despite the high prevalence of NVP, the therapy remains inconsistent and delayed.

## 1. Introduction

Nausea and vomiting in pregnancy (NVP) affect 50–90% of pregnant women and are common in early gestation with prevalence varying according to ethnicity and demographic characteristics [1,2,3]. NVP is often underestimated and impacts pregnant women’s health [4]. A variable rate of NVP will progress to hyperemesis gravidarum (HG) [5]. HG can cause severe dehydration and an imbalance of electrolytes that could lead to malnutrition and significant maternal and fetal morbidity [6]. Hormone changes have been detected historically as the etiology of NVP and HG and, in particular, the rise in human chorionic gonadotropin (hCG) [7]. The etiology is multifactorial, and emerging evidence attributes a crucial role to the pathways of growth differentiation and inflammatory pathways [8,9]. Several authors considered a correlation between NVP and low birth weight and preterm delivery [10,11]. NVP adversely affects the mother’s quality of life, quality of sleep, nutrition, and psychological status [9,12,13,14]. NVP is a major cause of hospitalization in early pregnancy [15]. In Italy, guidelines are lacking, and information on the incidence, clinical course and management of NVP remains fragmented [16]. Several national and international guidelines recommend the use of a scoring system to define the NVP level and optimize rules for diagnosis, hospitalization and treatment [17]. The clinical guidelines recommend various antiemetic regimens. Therapeutic options include doxylamine–pyridoxine as well as Ondansetron and Metoclopramide [18]. The Royal College of Obstetricians and Gynaecologists (RCOG) endorses stepwise medication therapy, and undertreatment is due to treatment inertia, fear of teratogenicity, and variation in clinical practice [19]. Suboptimal adherence to prescribed medications further complicates management difficulty [20]. However, compliance is influenced by numerous factors, like perceived effectiveness, fear of fetal damage, side effects of medications, health provider recommendations, and sociocultural beliefs. Given these critical lacunae, our prospective multicenter cohort study aimed to do the following: (a) Evaluate the NPV prevalence and severity during the three trimesters of pregnancy using the validated PUQE score system. (b) Gestational age at the first obstetric consult in relation to NVP severity and hospitalization rates for NVP. (c) Identify demographics, obstetric and socioeconomic variables associated with NVP occurrence and severity. (d) Identify clinical predictors of risk of NVP recurrence. (e) Evaluate current pharmacological therapies in terms of the timing and adequacy of treatment according to PUQE severity. (f) Clinician and patient reasons for treatment modification or discontinuation during the pregnancy. This evidence could guide the development of standardized protocols for NVP screening and management.

## 2. Materials and Methods

### 2.1. Study Design and Study Population

We conducted a prospective, multicenter, observational cohort study of pregnant women receiving prenatal care at eighteen obstetrics Hospitals or Universitaire-Hospitals in Italy, from 11 October 2022 to 1 November 2024. The centers were distributed in Italy: Northern Italy, ASST Fatebenefratelli-Sacco and University of Milan (Milan), S. Anna University Hospital (Turin), University of Ferrara (Ferrara), Azienda Ospedaliera Spedali Civili di Brescia (Brescia), Ospedali Riuniti (Livorno), and S. Maria della Misericordia University Hospital (Udine); Central Italy, AOU Careggi and University of Florence (Florence), San Salvatore Hospital and University of L’Aquila (L’Aquila), University Hospital of Chieti (Chieti), University of Siena (Siena), S. Pietro Fatebenefratelli Hospital (Rome) and Università degli Studi di Modena e Reggio Emilia (Modena); Southern Italy, University of Campania “Luigi Vanvitelli” (Naples), University of Naples “Federico II” (Naples), University of Foggia (Foggia), University of Cagliari (Cagliari), and San Carlo Hospital (Potenza). Every center aimed to consecutively enroll patients until the target of a total of 1000 patients. Follow-up was predefined at ≤13, 20–24, 30–34 and 37–38 weeks and was considered incomplete if at the patients did not follow the successive obstetric visit. We included all the pregnant women aged at least 18 years that attended the first prenatal visit within 13 weeks from the first day of the last menstruation and provided informed written consent. Pregnant women were followed until the post-partum period. We excluded the pregnant women that attended their prenatal visit after 13 weeks of gestational age, multiparity or with incomplete follow-up. Ethical approval was received from the Università degli studi della Campania Luigi Vanvitelli’s ethics committee (protocol number 0030746/i, 11 October 2022). This multicenter study was officially supported by the Italian Society of Gynecology and Obstetrics (SIGO) under the project title “Purity-Extended.” The study protocol was approved by SIGO prior to study enrollment. No Italian guidelines are actually present, and all centers treated the NVP independently, following the current literature recommendations [17,21].

### 2.2. Data Collection

After the first prenatal visit, eligible women who agreed to participate received a questionnaire for NVP evaluation. The NVP severity and frequency were assessed from the 1st to the predelivery period, with the PUQE. All pregnant women were prospectively followed at the prenatal control at ≤13, 20–24, 30–34 and 37–38 weeks of gestation. PUQE is a validated tool used to measure the severity of NVP [22]. The existence of NVP was described by physicians as dichotomous (yes/no). The intensity of NVP was assessed using the PUQE scale on the basis of three daily markers: daily nausea duration, frequency of retching and vomiting. The score ranges from 3 to 15, with 3 to 6 reflecting mild symptoms, 7 to 12 moderate symptoms, and 13 to 15 severe symptoms. The total PUQE score was obtained as the sum of these three item scores.

All centers collected prospective data during all the antenatal routine visits. The physicians recorded the PUQE and patients’ characteristics via Paper-and-Pen Interview (PAPI) methodology. Data were registered in an anonymous way. In each center, the database was stored in a password-protected file, accessible only by principal investigator. The anonymized datasets were transferred to the coordinating center in accordance with data protection and privacy laws. The following parameters were measured and documented. At first prenatal visit (≤13 weeks of pregnancy):

(a) Demographics and anthropometry data: age, ethnicity, country of residence, education level, employment status, pre-pregnancy BMI, habit of smoking.
(b) Obstetrical history: Parity and histories of nausea and vomiting in previous pregnancies (in multiparous women).
(c) NVP evaluation through severity analysis (PUQE score).
(d) Fetal and maternal pathologies included gestational diabetes, hypertension/preeclampsia, fetal growth restriction.
(e) Pre-visit therapy: pharmacological or non-pharmacological therapy adopted.
(f) Eventual patient hospitalization: incidence of admissions due to NVP/hyperemesis.

### 2.3. Statistical Analysis

On the basis of previous studies [16], we calculated that a sample size of 503 pregnant women is needed with an NVP prevalence of 70%, considering the Italian 369,944 pregnancies for years (2024 data), with a 95% confidence level and a 4% margin of error. Descriptive statistics were utilized to estimate the prevalence and the NVP severity and to summarize maternal demographic, anthropometric, and obstetric characteristics. Continuous variables were expressed as medians with interquartile ranges (IQRs) and compared between groups using the Wilcoxon rank-sum or Kruskal–Wallis tests, and categorical variables were presented as absolute and relative frequencies and compared using Fisher’s exact test. Univariate and multivariate logistic regression analyses were performed to identify sociodemographic and obstetric variables independently associated with NVP. Results were reported as odds ratios (ORs) with 95% confidence intervals (CIs). All tests were two-tailed, and *p* values < 0.05 were considered statistically significant. Statistical analyses were performed using R Studio, version 4.1.3 (R Foundation for Statistical Computing, Vienna, Austria).

## 3. Results

### 3.1. Subjects Enrolled and Characteristics

**Figure 1:**
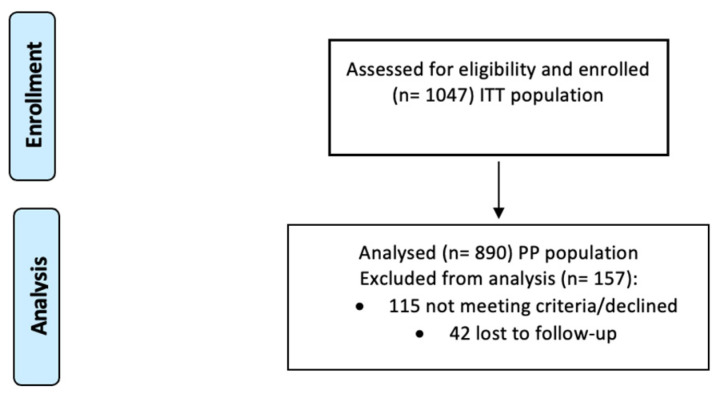
The included patient flowchart.

Among 1047 patients enrolled in the first trimester, 157 patients (15%) were excluded from the statistical analysis: 115 pregnant women (11%) for not meeting criteria (performing the first obstetric consult after the 13 weeks) or declining participation; 42 (4.0%) for incomplete follow-up (Figure 1).

A total of 890 pregnant women from the eighteen centers were included in the final analysis, 488 from the North-Center and 402 from the South of Italy. Demographics and baseline characteristics are presented in Table 1. Age and pre-pregnancy BMI did not differ between the two groups. The median maternal age was 32 years (29.0–36.0) for pregnant women without NVP and 32.9 years (30.9–34.8) among the NVP group. Pre-pregnancy BMI showed a median of 23.0 (20.0–26.6) and 22.8 (21.7–24.3) in the two groups (*p* = 0.22, Table 1). The majority of patients included were Caucasian (86.9% without NVP and 83.1% with NVP; *p*-value 0.19). Educational level, regional pregnant distribution and employment status were comparable between groups, with no statistical difference. Marital status showed a small difference between groups (*p* = 0.04), with a higher proportion of partnered or married women in the NVP group (94.7% vs. 90.6%; Table 1). Parity, first gestational age at the first visit and smoking before pregnancy did not show statistical differences (Table 2). Further, 50.2% of the pregnant women were nullipara in the NVP group (Table 2). The first obstetric consult was performed around 9.3–9.2 weeks of gestation. Smoking before pregnancy did not differ between groups (*p* = 0.94), but smoking during pregnancy was less frequent among women with NVP (3.9% vs. 7.1%; *p* = 0.04, Table 2). Current NVP showed a high rate of previous pregnancy with NVP history (63.2% vs. 36.8%, *p* < 0.001). The prevalence of pregnancy complications (gestational diabetes, gestational hypertension/preeclampsia or fetal growth restriction) did not differ significantly in the two groups (*p* = 0.24, Table 2).

### 3.2. NVP Severity and Pharmacological Treatment

**Figure 2:**
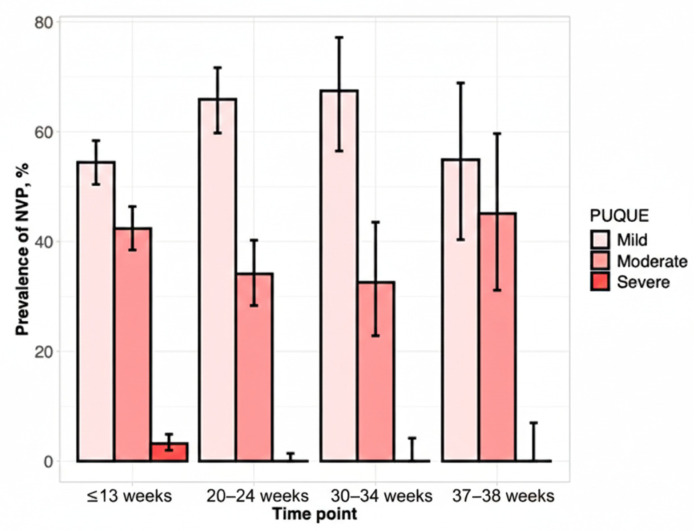
Clinical NVP severity categorized with the PUQE score.

Of the total pregnant women included in our study, 70% (623/890) showed NVP signs and symptoms. Clinical NVP severity was categorized with the PUQE score: 339 (54.4%, 339/623) mild, 264 (42.3%, 264/623) moderate, and 20 (3.2%) severe NVP (Table 3, Figure 2).

The hospitalization rate was 2.41% (15/623). Nulliparity was more frequent in mild cases (57.2%) than in moderate (44.3%) and severe (10.0%) cases (*p* < 0.001). Pluripara was associated mainly with the severe and mild PUQUE scores, 90% and 55.7%, respectively, and 42.8% for the mild symptoms (*p* < 0.001, Table 3). Severe forms acceded to the first obstetric consult late, with a gestational age of 11.0 and 9.7 weeks for the severe and moderate PUQUE score (*p* < 0.001, Table 3). Mild symptoms had a low median gestational age of 9.0 (7.8–11.1, *p* < 0.001, Table 3). Previous pregnancy with NVP showed an association with moderate and severe symptoms (72.1% and 77.8%, *p*-value < 0.001, Table 3). Pharmacological treatment was prescribed after the first obstetric visit to 50.7% of mild, 67.0% of moderate, and 50.0% of severe cases (*p* < 0.001). Further, 264 pregnant women (42.4%) did not receive pharmacological treatment and received only non-pharmacological and/or dietary recommendations. The main pharmacological treatment was the doxylamine succinate with pyridoxine, which was prescribed to all PUQUE severity subgroups: 44.2% mild, 58.7% moderate and 50% severe cases. Use of doxylamine succinate with pyridoxine hydrochloride increased with PUQE score severity (*p* < 0.001, Table 3). D2R antagonists were prescribed to 6.5% and 8.3% of the mild and moderate symptom groups.

### 3.3. Predictors of NVP and Daily Dosage Therapy

**Figure 3:**
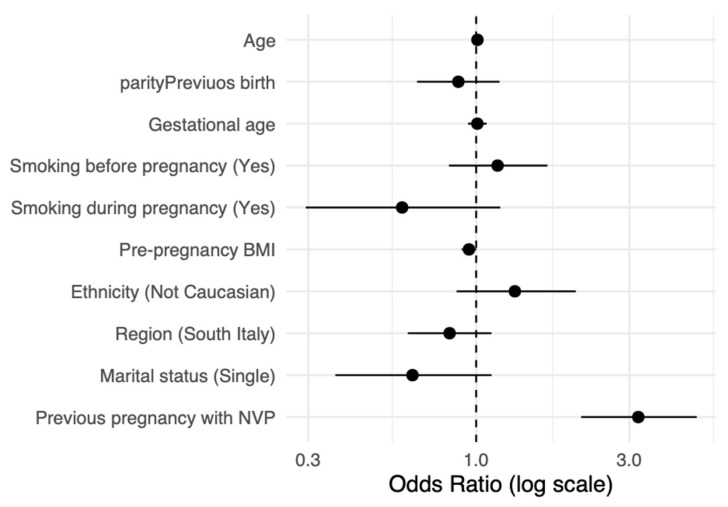
Multivariable logistic regression of NVP predictors.

To explore the predictors of NVP, we performed a multivariable logistic regression, shown in Figure 3.

NVP was associated with previous NVP history (OR = 3.20, 95% CI 2.12–4.83, *p* < 0.001), substantiating a strong relationship with NVP recurrence and personal predisposition. Pre-pregnancy BMI (OR = 0.95, 95% CI 0.90–1.00, *p* = 0.052) and maternal age (OR = 1.01, 95% CI 0.97–1.05, *p* = 0.655, Figure 3) did not show significant associations. Non-Caucasian ethnicity (OR = 1.32, 95% CI 0.86–2.03, *p* = 0.202), smoking before pregnancy (OR = 1.17, 95% CI 0.83–1.65, *p* = 0.393), and parity (OR = 0.88, 95% CI 0.65–1.18, *p* = 0.397) did not show a significant association with NVP. Similarly, single marital status (OR = 0.63, 95% CI 0.36–1.10, *p* = 0.109), south region (OR = 0.83, 95% CI 0.62–1.12, *p* = 0.213), gestational age at first visit (OR = 1.01, 95% CI 0.94–1.08, *p* = 0.805) and current smoking (OR = 0.59, 95% CI 0.29–1.18, *p* = 0.134, Figure 3) were associated with lower odds ratios. We explored the daily dosages of the main antiemetic therapy prescribed: doxylamine succinate with pyridoxine hydrochloride. One capsule had 10 mg of doxylamine succinate with 10 mg of pyridoxine hydrochloride. The majority of pregnant women with mild (119/148) or moderate (56/159) NVP had a prescription of one or two capsules per day (12.2% and 68.2% vs. 5.7% and 42.1%, respectively), with no statistical differences (*p* = 0.72, Table 4). Conversely, higher-dosage (3–5 capsules/day, *p* < 0.001) prescriptions increased with PUQUE severity score: 4.1% and 14.9% of mild scores compared with 20.8% and 30.2% of moderate NVP, and up to 40.0% and 30.0% of severe symptoms (Table 4).

### 3.4. Changes in Therapy During the Different Obstetric Visits

At the second obstetric consultation (Table 5 and Figure 3), the majority of pregnant women continued their initial therapy, with no statistical differences in dosage adjustments (59.4% of mild vs. 59.1% of moderate, *p* = 0.99, Table 5). Reasons for therapy interruption showed a statistical difference (*p* < 0.001, Table 5), with 10.0% of mild NVP interrupting treatment due to symptom acceptability. Moderate NVP cases interrupted the therapy due to lack of efficacy or fear of adverse effects (*p* < 0.001, Table 5). New prescriptions did not differ between groups (*p* = 0.49, Table 5). Analyzing the third visit (Table 6), 60.3% of mild and 32.1% of moderate cases continued the therapy (*p* = 0.03); however, reductions were more frequent in the mild cases, 71.4% versus 66.7%. Treatment interruptions were uncommon (8.6–14.3%, *p* = 0.66, Table 6). Women with mild NVP maintained unchanged therapy (Table 6). At the last obstetric visit (Table 7), therapeutic behavior was consistent with previous trends. Continuation of therapy was reported in 39.3% of mild and 26.1% of moderate NVP cases (*p* = 0.34), indicating progressive discontinuation as symptoms resolved. Therapy interruption occurred rarely (10.7% and 0%, respectively, mild and moderate, *p*-value 0.45). Across all third and fourth consults, no significant differences were observed in as-needed medication use (Table 6 and Table 7).

## 4. Discussion

Our findings, from a large Italian multicentric study, evidenced that 70% of pregnant women experience pregnancy-related nausea and vomiting from the beginning of the pregnancy. NVP was highly prevalent, with approximately half of the included pregnant women reporting mild to severe NVP. Our NVP prevalence aligns with another recent Italian study, which explored NVP prevalence in Italy, analyzing a population of 232 Italian pregnant women [16,22]. We evidenced a strong association between a previous history of NVP and recurrence of NVP, with a threefold increased risk. Several biological mechanisms can explain our findings [18]. Susceptibility through heredity means might also play a role, as our study reports a significant correlation between history of NVP in past pregnancy and symptomatology at present [23]. Heredity origin was explored with recent articles that identified gene variants implicated in the placenta and hormone functions, like GDF15, IGFBP7, GFRAL and PGR, in NVP and hyperemesis gravidarum [24]. This supports a hypothesis of a hereditary and placental-mediated etiology component in NVP [24]. Considering our data, demographic, anthropometric and socioeconomic characteristics were comparable in pregnant women with and without symptoms. No statistical associations were evidenced for maternal age, pre-pregnancy BMI, parity, smoking, or regional origin. After the first obstetric visit, around half of the pregnant women received pharmacological treatment. Interestingly, we evidenced late access to the first prenatal care for the moderate and severe cases. This delay could explain the increased rate of severe NVP forms, probably related to a delay in the correct management of these patients. In addition, the hCG levels during early pregnancy have been involved in the pathogenesis of NVP ever since, and maximal symptomatology has been found to relate to maximal hCG levels during 9–12 weeks of gestation [24]. Pharmacologic treatment was prescribed in around half of symptomatic women and increased across severity levels. The combination of doxylamine succinate and pyridoxine hydrochloride represented the principal prescription (44.2% of mild, 58.7% of moderate, and 50.0% of severe cases), especially for severe symptoms, where it represented a unique therapy. D2-receptor antagonists were prescribed rarely.

Our analysis also evidenced an inverse association between current smoking and NVP, probably due to smoking aversion in patients with NVP. Smoking could influence maternal nausea and induce the patient to avoid smoking odors during pregnancy. In accordance with the literature, severe forms and hyperemesis gravidarum with hospitalization were low (2.4%) [25]. Considering the literature, our findings confirm the multifactorial nature of NVP and its recurrence in patients with previous NVP. Different studies evidenced an increased risk of NVP with an increased gravidity and multiparity [26,27]. Our data lack significant associations with ethnicity, probably due to the few Asian or Black patients included [22]. Regarding the pharmacologic treatment, our data reflect real-word clinical practice. Doxylamine–pyridoxine represents the first-line agent endorsed by obstetric societies [20,28]. The prescription rate of 57.6% in our cohort suggests increasing physician confidence in the safety of medical treatment [29]. The observed late prenatal consult among women with severe symptoms underscores the necessity of early counseling and timely therapeutic intervention for this condition. Further, 68.2% of patients with mild symptoms received two capsules/day; those with medium-level symptoms received three (20.6%) or even four (30%) capsules daily with increasing frequency. Of particular concern, a third of patients with severe NVP received five capsules daily. This kind of dose approach supports individualized treatment and introduces consideration about pharmacokinetics and tolerance for higher dose regimens in pregnancy.

After treatment began, considering the treatment adherence, significant differences were found. On the second prenatal visit (14–20 weeks), half of women had fallen out of therapy because of the spontaneous resolution of symptoms. This increased to 72.7% by the third visit (at 28–32 weeks), aligning with the natural course of NVP. Few patients at the second visit and none at any subsequent visits reported cessation of treatment because of fear of ineffectiveness and fear of teratogenic effects, a common situation, as cited before. At the fourth visit, 32.1% of participants reduced the therapy dose, while no dose increases were reported. This trend is consistent with clinical practice, where medication tapering is commonly implemented in response to symptomatic improvement. However, a small group of patients continued or intensified therapy, highlighting the heterogeneity of the NVP course. Our analysis evidenced the undertreatment of NVP with adjunctive or second-line drugs. Despite guideline recommendations advocating for a stepped pharmacologic approach in women who do not respond to first-line therapy, combined regimens or additional antiemetics were infrequently observed. This may reflect clinician hesitation, limited medication availability, or patient preference, ultimately representing missed opportunities for effectively managing severe NVP cases.

From a public health perspective, the implications of our findings are of considerable consequence. First, clinical practice tends to underestimate moderate–severe NVP cases. Second, the tapering of therapy over time reflects the natural history of NVP and supports both the safety and feasibility of pharmacologic treatment. Third, different dosage and symptom duration underscores the need for tailored treatment regimens. The limitations of this study include the absence of a control group without therapy, potential recall bias in the reporting of symptom onset, and incomplete data on dietary intake and quality-of-life parameters. In addition, despite the large sample in our study, severe NVP cases were only twenty, and this limited the generalizability of our conclusion for the severe NVP form. Additional prospective studies with biochemical markers, dietary evaluation, and neonatal outcomes will be required to characteristically describe the impact of therapy adherence in maternal–fetal well-being. Specifically, studies are also needed for the severe NVP form.

Overall, this study offers a clinically relevant and accurate characterization of NVP management, highlighting symptom intensity. Moreover, it enlightens the central role of doxylamine–pyridoxine as first-line therapy and the dynamic, pregnancy-specific nature of treatment adherence. These findings support the need for improved provider education, thoughtful prescribing practices, and structured follow-up to optimize outcomes in pregnancy-related NVP. Considering the NVP impact on maternal quality of life, and its potential to produce maternal dehydration, malnourishment, as well as psychological distress in severe forms, early recognition and active management are necessary [30,31]. Correct counseling and correct screening, especially for multipara patients and early initiation of antiemetic treatment when indicated, are our recommendations [17,32]. Moreover, future studies are needed to assess the severe NVP forms and to evaluate the other factors that impact medical treatment.

## 5. Conclusions

Nausea and vomiting during pregnancy are common among Italian pregnant women and are typically mild to moderate in severity. Undertreatment remains frequent. Standardized assessment and early diagnosis are needed to ensure a correct intervention.

## Figures and Tables

**Table 1:** Demographic characteristics of the pregnant women included.

	Nausea and/or Vomiting During Pregnancy		
Characteristic	No, N = 267 ^1^	Yes, N = 623 ^1^	*p*-Value ^2^
Age	32.0 (29.0, 36.0)	32.9 (30.9, 34.8)	0.34
Pre-pregnancy BMI	23.0 (20.0, 26.0)	22.8 (21.7, 24.3)	0.22
Ethnicity			0.19
Caucasian	232.0 (86.9%)	518.0 (83.1%)	
Not Caucasian	35.0 (13.1%)	105.0 (16.9%)	
Education level			0.18
Bachelor’s or Master’s degree	79.0 (29.6%)	212.0 (34.0%)	
Lower secondary education	51.0 (19.1%)	105.0 (16.9%)	
Master’s degree	16.0 (6.0%)	56.0 (9.0%)	
Upper secondary education	121.0 (45.3%)	250.0 (40.1%)	
Occupation			0.54
Employed	174.0 (65.2%)	391.0 (62.8%)	
Unemployed	93.0 (34.8%)	232.0 (37.2%)	
Marital status			0.04
Partner or Married	242.0 (90.6%)	590.0 (94.7%)	
Single	25.0 (9.4%)	33.0 (5.3%)	
Region			0.06
Northen/Central Italy	133.0 (49.8%)	355.0 (57.0%)	
South Italy	134.0 (50.2%)	268.0 (43.0%)	

^1^ Median (Q1, Q3) or Frequency (%) ^2^ Wilcoxon rank sum test; Fisher’s exact test.

**Table 2:** Obstetric characteristics of the pregnant women included.

	Nausea and/or Vomiting During Pregnancy		
Characteristic	No, N = 267 ^1^	Yes, N = 623 ^1^	*p*-Value ^2^
Parity			0.31
Nulliparity	124.0 (46.4%)	313.0 (50.2%)	
Previous birth	143.0 (53.6%)	310.0 (49.8%)	
Gestational age	9.3 (7.4, 11.8)	9.20 (7.8, 11.5)	0.75
Smoking before pregnancy			0.94
No	189.0 (70.8%)	438.0 (70.3%)	
Yes	78.0 (29.2%)	185.0 (29.7%)	
Smoking during pregnancy			0.04
No	248.0 (92.9%)	599.0 (96.1%)	
Yes	19.0 (7.1%)	24.0 (3.9%)	
Pregnancy complications			0.24
Fetal growth restriction	21.0 (7.9%)	34.0 (5.5%)	
Gestational Diabetes	23.0 (8.6%)	76.0 (12.2%)	
Gestational Hypertension/Preeclampsia	6.0 (2.2%)	18.0 (2.9%)	
No complications	217.0 (81.3%)	495.0 (79.5%)	
Previous pregnancy with nausea and/or vomiting			<0.001
No	93.0 (65.0%)	114.0 (36.8%)	
Yes	50.0 (35.0%)	196.0 (63.2%)	
Nullipara	124	313	

^1^ Median (Q1, Q3) or Frequency (%) ^2^ Fisher’s exact test; Wilcoxon rank sum test.

**Table 3:** Clinical NVP severity following the PUQE score.

	PUQUE Severity Score			
Characteristic	Mild, N = 339 ^1^	Moderate, N = 264 ^1^	Severe, N = 20 ^1^	*p*-Value ^2^
Parity				<0.001
Nulliparity	194.0 (57.2%)	117.0 (44.3%)	2.0 (10.0%)	
Previous birth	145.0 (42.8%)	147.0 (55.7%)	18.0 (90.0%)	
Gestational age	9.0 (7.8, 11.1)	9.7 (7.8, 11.7)	11.0 (10.1, 11.9)	<0.001
Previous pregnancy with nausea and/or vomiting				<0.001
No	69.0 (47.6%)	41.0 (27.9%)	4.0 (22.2%)	
Yes	76.0 (52.4%)	106.0 (72.1%)	14.0 (77.8%)	
Nullipara	194	117	2	
Pharmacological treatment				<0.001
No	167.0 (49.3%)	87.0 (33.0%)	10.0 (50.0%)	
Yes	172.0 (50.7%)	177.0 (67.0%)	10.0 (50.0%)	
Reference pharmacological treatment				<0.001
D2R antagonists	22.0 (6.5%)	22.0 (8.3%)	0.0 (0.0%)	
Doxylamine succinate with Pyridoxine hydrochloride	150.0 (44.2%)	155.0 (58.7%)	10.0 (50.0%)	
No treatment	167.0 (49.3%)	87.0 (33.0%)	10.0 (50.0%)	

^1^ Median (Q1, Q3) or Frequency (%) ^2^ Fisher’s exact test; Kruskal–Wallis rank sum test.

**Table 4:** Daily dosage of doxylamine succinate with pyridoxine hydrochloride.

	PUQUE Severity Score			
Dosage (cps/die)	Mild, N = 148 ^1^	Moderate, N = 159 ^1^	Severe, N = 10 ^1^	*p*-Value ^2^
1 cps/die	18 (12.2)	9 (5.7)	0 (0.0)	Ref
2 cps/die	101 (68.2)	67 (42.1)	1 (10.0)	0.72
3 cps/die	6 (4.1)	33 (20.8)	4 (40.0)	<0.001
4 cps/die	22 (14.9)	48 (30.2)	2 (20.0)	<0.001
5 cps/die	0 (0.0)	0 (0.0)	3 (30.0)	<0.001
As needed	1 (0.7)	2 (1.3)	0 (0.0)	0.54

^1^ Number (%) ^2^ Fisher’s exact test.

**Table 5:** Therapy at the second obstetric appointment.

Second Consult	PUQUE Severity Score		
Characteristic	Mild, N = 170 ^1^	Moderate, N = 88 ^1^	*p*-Value ^2^
Continued therapy	101 (59.4)	52 (59.1)	0.99
Increased	11 (10.9)	6 (11.5)	
Reduced	45 (44.6)	9 (17.3)	
Unchanged	42 (41.6)	37 (71.2)	
As needed	2 (2.0)	0 (0.0)	
Interrupted therapy	22 (12.9)	12 (13.6)	<0.001
Symptom tolerated	17 (10.0)	0 (0.0)	
Lack of efficacy	1 (0.6)	5 (5.7)	
Fear	0 (0.0)	3 (3.4)	
New prescription	4 (2.4)	4 (4.5)	0.49
No treatment at first visit	47 (27.6)	24 (27.2)	0.91

^1^ Numbers (Frequency, %) ^2^ Chi-squared (χ^2^) test.

**Table 6:** Therapy at the third obstetric visit.

Third Consult	PUQUE Severity Score		
Characteristic	Mild, N = 58 ^1^	Moderate, N = 28 ^1^	*p*-Value ^2^
Continued therapy	35 (60.3)	9 (32.1)	0.03
Increased	0 (0.0)	0 (0.0)	
Reduced	25 (71.4)	6 (66.7)	
Unchanged	10 (28.6)	3 (33.3)	
As needed	0 (0.0)	0 (0.0)	
Interrupted therapy	3 (8.6)	4 (14.3)	0.66
Symptom tolerated	2 (66.7)	0 (0.0)	
Lack of efficacy	1 (33.3)	2 (50.0)	
Fear	0 (0.0)	2 (50.0)	
New prescription	0 (0.0)	0 (0.0)	
Discontinued treatment at previous visit	5 (8.6)	4 (14.3)	0.48
No treatment at first visit	15 (25.9)	11 (39.3)	0.24

^1^ Numbers (Frequency, %) ^2^ Chi-squared (χ^2^) test.

**Table 7:** Therapy at the last obstetric visit.

Fourth Consult	PUQUE Severity Score		
Characteristic	Mild, N = 28 ^1^	Moderate, N = 23 ^1^	*p*-Value ^2^
Continued therapy	10 (39.3)	6 (26.1)	0.34
Increased	0 (0.0)	0 (0.0)	
Reduced	0 (0)	0 (0.0)	
Unchanged	10 (100.0)	6 (100.0)	
As needed	0 (0.0)	0 (0.0)	
Interrupted therapy	3 (10.7)	0 (0.0)	0.45
Symptom tolerated	0 (0.0)	0 (0.0)	
Lack of efficacy	1 (33.3)	0 (0.0)	
Fear	2 (66.6)	0 (0.0)	
Change prescription	0 (0.0)	0 (0.0)	
Discontinued treatment at previous visit	2 (7.1)	6 (26.1)	0.34
No treatment at first visit	12 (42.9)	11 (47.8)	0.90

^1^ Numbers (Frequency, %) ^2^ Chi-squared (χ^2^) test.

## Data Availability

All data generated or analyzed during this study are available upon reasonable request.

## Abbreviations

The following abbreviations are used in this manuscript:

NVP	Nausea and Vomiting of Pregnancy
HG	Hyperemesis Gravidarum
PUQE	Pregnancy-Unique Quantification of Emesis
BMI	Body Mass Index
OR	Odds Ratio
CI	Confidence Interval
hCG	Human Chorionic Gonadotropin
RCOG	Royal College of Obstetricians and Gynaecologists
SIGO	Italian Society of Gynecology and Obstetrics
D2R	Dopamine D2 Receptor
IQR	Interquartile Range
PAPI	Paper-and-Pen Interview
